# Metastatic Ureteral Involvement of Non-Small Cell Lung Cancer

**DOI:** 10.1155/2011/394326

**Published:** 2011-03-30

**Authors:** Koichi Kodama, Tetsuya Imao, Kazuto Komatsu

**Affiliations:** ^1^Department of Urology, Toyama City Hospital, 2-1 Imaizumihokubu-cho, Toyama 939-8511, Japan; ^2^Department of Urology, Nagano Red Cross Hospital, 5-22-1 Wakasato, Nagano 830-8582, Japan; ^3^Department of Urology, Fukui Red Cross Hospital, 2-4-1 Tsukimi, Fukui 918-8501, Japan

## Abstract

Metastases from a variety of malignant tumors can involve the ureters, but ureteral involvement by lung cancer is extremely rare and usually described at autopsy. We report a rare case of a 76-year-old man who presented with a three-month history of right flank dullness and was noted to have a nonhomogeneous retroperitoneal mass with hydronephrosis of the right kidney on computed tomography of the abdomen. Computed tomography of the thorax showed a nodule in the lower lobe, measuring 3 × 2 cm, in the right lung. After excluding the presence of other primary tumors and metastases, we reached a final diagnosis of solitary retroperitoneal metastasis of adenocarcinoma of the lung. Although rare, in patients of non-small cell lung cancer, presence of hydronephrosis should alert the physician to the possibility of metastasis.

## 1. Introduction

Lung cancer is the most common cause of cancer death for both genders. At the time of diagnosis, only 20% of all lung cancer patients will have local disease, while 55% will have distant metastatic disease [1]. The brain is the most common site of metastases of lung cancer followed by the bones, liver, and adrenal glands [2]. However, ureteral obstruction caused by metastases of lung cancer is extremely rare. Here we report a unique case of metastatic ureteral involvement of non-small cell lung cancer which was the first clinical evidence of the underlying malignancy.

## 2. Case Presentation

**Figure 1:**
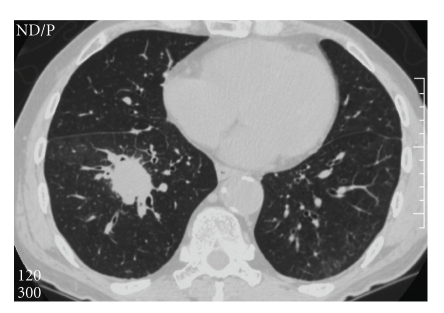
Computed tomography of the thorax showed a soft tissue density nodule with spiculated margins and vascular convergence in the lower lobe, measuring 3 × 2 cm, in the right lung.

A 76-year-old man presented with a three-month history of right flank dullness. He had not had any respiratory symptoms, and no malignancies had been diagnosed. He did not smoke tobacco. Physical examination results were mostly normal. Computed tomography (CT) of the abdomen showed a non-homogeneous retroperitoneal mass with hydronephrosis of the right kidney. CT of the thorax showed a soft tissue density nodule with spiculated margins and vascular convergence in the lower lobe, measuring 3 × 2 cm, in the right lung (Figure 1). These morphologic characteristics on CT scans were suspicious for primary lung cancer. CT of the head and bone scan were negative for metastases. Laboratory data, including those for prostate-specific antigen, *α*-fetoprotein, *β*-human chorionic gonadotropin, carcinoembryonic antigen, and carbohydrate antigen 19-9, were within normal limits except for elevated serum creatinine of 1.4 mg/dL (normal, 0.6 mg/dL–1.1 mg/dL). Urinalysis results were also normal.

**Figure 2:**
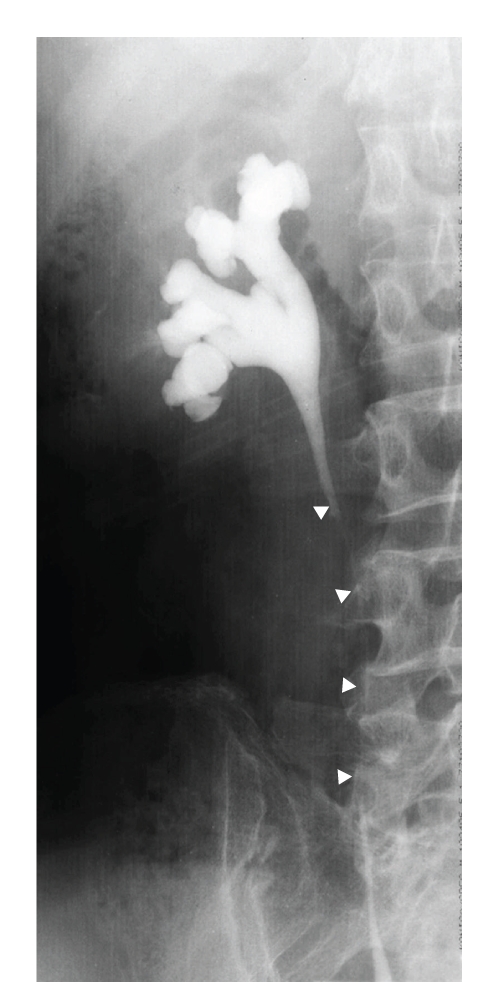
A right retrograde ureterogram showed a continuous extrinsic obstruction of the middle third of the ureter (arrowheads).

**Figure 3:**
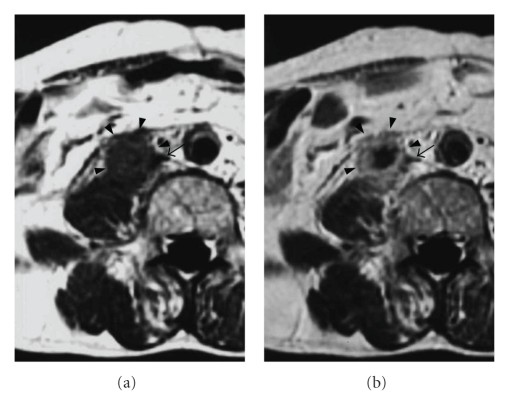
(a) T1-weighted magnetic resonance images demonstrated a retroperitoneal mass (arrowheads) with compression of the inferior vena (arrow). (b) Contrast-enhanced T1-weighted image showed the mass in a ring-like fashion.

**Figure 4:**
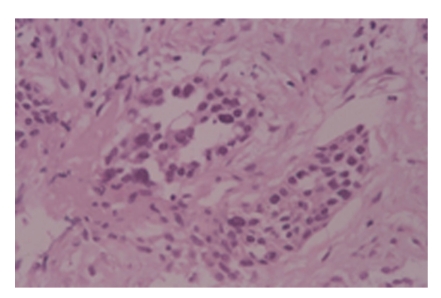
Pathological examination of the retroperitoneal mass indicated a poorly differentiated adenocarcinoma with fibrosis.

A right retrograde ureterogram showed a continuous extrinsic obstruction, 7 cm in length, of the middle third of the ureter (Figure 2). The results of cytologic assessment of urine specimens collected from the right ureter were normal. T1-weighted magnetic resonance images demonstrated a retroperitoneal mass, measuring 3 × 3 × 5 cm, with compression of the inferior vena cava (Figure 3(a)). Enhancement by gadolinium-diethylenetetraminepentaacetic acid of the T1-weighted images showed a high-intensity rim (Figure 3(b)). Fiberoptic bronchoscopy showed that a luminal mass caused obstruction of the bronchi and was used for brushing of the lesion for cytologic evaluation of the specimens, which showed the tumor to be a poorly differentiated adenocarcinoma. CT-guided fine needle biopsy of the retroperitoneal mass was performed, and the pathological examination indicated a poorly differentiated adenocarcinoma with fibrosis (Figure 4). The pathological features were consistent with metastasis of adenocarcinoma of the lung.

After excluding the presence of other primary tumors and metastases, we reached a final diagnosis of solitary retroperitoneal metastasis of adenocarcinoma of the lung, stage IV (T2N0M1). Subsequent occurrence of many small lung nodules, which may be consistent with metastatic disease, was not observed. Chemotherapy was initiated, but the patient died as a result of compression of the inferior vena cava by the retroperitoneal metastasis 6 months later after the diagnosis.

## 3. Discussion

Hematogenous or lymphatic metastases from a distant primary neoplasm to the ureter are encountered infrequently. Babaian et al. reported 37 (0.3%) of 11,689 patients with malignant disease had histologically proved metastatic lesions to the ureter in a large series of autopsies [3]. The rarity of their occurrence probably reflects the segmental distribution of the blood and lymphatic vessels in the ureter and periureteral sheath, resulting in the absence of continuous longitudinal networks [4]. Secondarily, the lymph drainage of the ureter of the lower portion of the ureter is downward and counterdirectional to that of the pelvic organ [5]. The primary sites include stomach, colon, uterine cervix, breast, skin, prostate, lung, and adrenal gland. In the autopsy data, metastases to the ureter was found in 2 (0.2%) of 1281 lung cancer patients [3].

In the majority of cases, the presence of ureteral metastases is not either diagnosed or suspected prior to death. Early symptoms of ureteral metastases may be masked by the overbearing presence of the primary tumor or other metastases, or by the generally poor condition of the patient. In the present case, metastatic ureteral involvement of non-small cell lung cancer was the first clinical evidence of the underlying malignancy. 

Radiographic findings of extrinsic ureteral obstruction due to metastatic diseases depend on the pattern of the tumoral spread: hematogenous submucosal/mucosal metastasis, hematogenous adventitia metastases spreading along periureteral vessels, scirrhous metastatic spread along periureteral vessels, or metastatic spread into lymph nodes with perinodal desmoplastic reaction [6]. The present case demonstrated the scirrhous spread pattern, which is seen especially in adenocarcinomas of the prostate, stomach, and colon [7–9].

Accurate determination of the extent of disease is critical in patients with lung cancer, as it has implications for both prognosis and treatment. The choice of surgical biopsy technique for diagnostic confirmation depends on many factors, for example, the size and anatomic location of the mass, and the overall estimated risk of malignancy. It should also be considered that the metastatic cells are usually dispersed diffusely in fibrotic plaque. In our case, multiple fine-needle aspiration biopsies of the retroperitoneal mass under CT guidance could establish the diagnosis and avoid unnecessary surgical exploration. 

In patients of lung cancer, presence of hydronephrosis should alert the clinician to the possibility of metastasis and subsequent evaluation.
